# High Concordance between Vaginal Samples and Cervical Samples of Human Papillomavirus in Women Living with HIV in Rwanda

**DOI:** 10.1186/s12879-025-10840-7

**Published:** 2025-04-15

**Authors:** Schifra Uwamungu, Maria Andersson, Bethelehem Nigussie Zewdie, Claude Mambo Muvunyi, Emile Bienvenu, Bengt Hasséus, Daniel Giglio

**Affiliations:** 1 Department of Pharmacology, Sahlgrenska Academy at the University of Gothenburg, Gothenburg, Sweden; 2 Present Address: Department of Biomedical Laboratory Sciences, School of Health Sciences, College of Medicine and Health Sciences, University of Rwanda, Kigali, Rwanda; 3 Present Address: Department of Infectious Diseases, Institute of Biomedicine, Sahlgrenska Academy at the University of Gothenburg, Gothenburg, Sweden; 4 Department of Pathology, Armauer Hansen Research Institute, P.O. Box 1005, Addis Ababa, Ethiopia; 5 Department of Clinical Biology, School of Medicine and Pharmacy, College of Medicine and Health Sciences, University of Rwanda, Kigali, Rwanda; 6 Rwanda Biomedical Center, Kigali, Rwanda; 7 Rwanda Food and Drugs Authority, Kigali, Rwanda; 8 Department of Pharmacy, School of Medicine and Pharmacy, University of Rwanda, Kigali, Rwanda; 9 Department of Oral Medicine and Pathology, Institute of Odontology, Sahlgrenska Academy at the University of Gothenburg, Gothenburg, Sweden; 10 Clinic of Oral Medicine, Public Dental Service, Gothenburg, Sweden; 11 Department of Oncology, Institute of Clinical Sciences, Sahlgrenska Academy at the University of Gothenburg, Blå Stråket 2, 41345 Gothenburg, Sweden; 12 Department of Oncology, Sahlgrenska University Hospital, Gothenburg, Sweden

**Keywords:** Human papillomavirus, HIV, Cervical cancer, Screening, Self-sampling, Rwanda

## Abstract

**Background:**

Chronic infection by human papillomavirus (HPV) is necessary for the development of almost all cervical cancers. The study assessed the prevalence of vaginal and cervical HPV infections in women living with HIV (WLWH) in Rwanda and assessed the performance of vaginal HPV screening to detect cervical HPV infections.

**Methods:**

HIV-positive women (*N* = 413) attending the HIV clinic at the University Teaching Hospital of Kigali, Kigali, Rwanda, were interviewed and vaginal and cervical swab samples for HPV testing and a pap smear sample were taken. RT-PCR was performed to detect twelve high-risk (HR)-HPVs (HPV16, 18, 31, 33, 35, 39, 45, 51, 52, 56, 58, and 59 and two low-risk (LR)-HPVs (HPV6 and 11) and conventional cytology was performed.

**Results:**

Vaginal HR-HPV/LR-HPV infections occurred in 39.2%/4.4% of women, while cervical HR-HPV/LR-HPV infections occurred in 38.3%/4.8% of women. HPV58 (14.3%/13.5%), HPV52 (12.5%/12.4%), HPV51 (12.9%/11.7%) and HPV16 (9.9%/11.3%) were the most prevalent HPV infections in the vagina/cervix and one third of HPV-positive women were infected with more than one HPV type. Normal cytology was less common in women with cervical HR-HPV infections compared to HPV-negative women (75.8% and 87.9%, respectively, *p* = 0.032). The type-specific HPV agreement for all HPV types between vaginal and cervical samples was 98.4% (kappa: 0.82 ± 0.02; *p* < 0.0001). Screening for vaginal HPVs gave a sensitivity of 83.5% (confidence interval: 78.4–87.7), specificity of 99.1% (98.8–99.3%), positive predictive value of 81.6% (77.0–85.5%) and negative predictive value of 99.2% (99.0–99.4%) for cervical HPV infections.

**Conclusions:**

HR-HPVs are common in WLWH in Rwanda. Vaginal HPV testing may potentially be used to simplify cervical cancer screening in the future in Rwanda.

**Supplementary Information:**

The online version contains supplementary material available at 10.1186/s12879-025-10840-7.

## Introduction

Cervical cancer is responsible for the majority of cancer-related death in women living in Sub-Saharan Africa [1–3]. Chronic infection by human papillomavirus (HPV) is necessary for the development of almost all cervical cancers [4]. Previous studies from our group have shown that cervical HPV infections are very common in Rwanda, particularly in risk groups such as women living with HIV (WLWH) [5–9]. In western countries, HPV screening is commonly used to identify women at risk of developing cervical cancer [10, 11]. HPV screening is not available for the majority of people living in Sub-Saharan Africa. Instead, cervical cancer screening relies on other techniques to detect cervical lesions such as visual inspection with acetic acid (VIA), visual inspection with Lugol’s iodine (VILI), VIA/VILI with magnification and Pap smear tests [12, 13]. The cervical screening method used depends on factors such as availability, costs, infrastructure, health care provider training and cultural acceptability.

There is an essential need to simplify screening techniques to identify women at risk of cervical cancer in developing countries. Studies outside of Africa have shown a good concordance between vaginal and cervical HPV infections [14–18]. It is also feasible for African women to perform vaginal self-sampling for HPV testing [5, 19–21]. However, if the HPV test is positive, the woman needs a second visit for follow-up, which makes HPV screening more logistically challenging, time-consuming and costly compared to a screen-and-treat approach such as VIA [22]. One step in the right direction to simplify HPV screening is to provide women home collection kits for HPV testing, which has been shown to be functional in Western countries and in Western low-resource settings [23, 24]. Currently, it is unclear whether this approach is feasible in Sub-Saharan countries. Notably, even if vaginal HPV triaging for cervical cancer screening is effective, there is evidence for vaginal tropism for certain phylogenetic HPV species, *e.g.*, for α3/α15, meaning vaginal HPV status may not fully represent cervical status [25, 26].

In our previous study in a 50-patient cohort of WLWH treated at the HIV clinic at the University Teaching Hospital of Kigali (CHUK), Kigali, Rwanda, we showed moderate to good concordance between vaginal HPV self-sampling and vaginal and cervical HPV samplings taken with the help from medical personnel [5]. In the current study we examined the prevalence of low-risk HPVs (α3; HPV6, HPV11) and high-risk HPVs (α5: HPV51; α6: HPV56; α7: HPV18, HPV39, HPV45, HPV59; α9: HPV16, HPV31, HPV33, HPV35, HPV52, HPV58) in vaginal and cervical samples in a new cohort of 413 women receiving treatment at the HIV clinic at CHUK [27]. We validated findings from our previous smaller study in this larger cohort of younger women and explored whether concordance between vaginal HPV and cervical HPV status depends on the HPV type.

## Materials and methods

A total of 413 HIV-positive women attending and receiving antiretroviral therapy (ART) at the HIV clinic, the University Teaching Hospital of Kigali (CHUK), Kigali, Rwanda, were recruited after providing informed consent between October to December 2021. Eligibility criteria for the study included being 21 years or older, diagnosed with HIV, seeking voluntary for HPV screening service and providing a signed informed consent form. Exclusion criteria were prior HPV vaccination, women diagnosed with cervical lesions and/or cancer before or at the time of inclusion, women with known or visibly present vaginal or cervical infection besides HPV at the time of inclusion, women who had not been engaged in sexual activity with other individuals, and women who, for any reason, were considered unable to comply with the study protocol.

A structured questionnaire (supplementary file) was administered to the participants, which included questions regarding their education, occupation, medical history, sexual and obstetric history, previous sexually transmitted infections and risk factors for HPV infection. Following the questionnaire, the physician took a swab sample from the vagina and one from the cervix for HPV testing utilizing the Aptima Multitest Swab (Hologic Inc., Marlborough, MA, USA). In addition, the physician took another swab from the vagina and one from the cervix where the swabs were put in RIPA buffer (Thermo Fisher, Rockford, Illinois, USA) for biomarker testing at the 2-year follow-up in a subsequent study. Pap smear samples were collected using a wood spatula and cyto-brush from the cervix. We decided to use pap smear test since at the start of the study, Thinprep pap test was not used in Rwanda. Samples collected for HPV testing were stored at room temperature. Women with abnormal cytology and/or high-risk (HR)-HPV positivity were referred from the HIV clinic to the gynecology department at CHUK.

### Real-time PCR for detection of HPVs

A real-time polymerase chain reaction (RT-PCR) assay was performed to target type-specific segments of the E6/E7 region for 12 high-risk (HPV16, 18, 31, 33, 35, 39, 45, 51, 52, 56, 58, and 59), two low-risk (HPV6 and 11) HPV types, and beta-globin, as previously described [28]. Nucleic acid was extracted from 200 µl of each specimen utilizing the DNA Isolation I kit and the MagNA Pure LC instrument (Roche Molecular, Mannheim, Germany). The extracted nucleic acids were eluted in a volume of 100 µl, and 5 µl were utilized for each RT-PCR reaction. The RT-PCR was performed in 8 parallel 20 µl reactions, containing oligonucleotides listed in Table S1, and Universal Mastermix (Applied Biosystems, Foster City, CA). The RT-PCR was run for 45 cycles (15 s at 95 °C, 60 s at 58 °C) following an initial 10 min denaturation at 95 °C in a QuantStudio 6 384-well system (Applied Biosystems, Waltham, MA, USA). To ensure the performance of each multiplex reagent mixture, pUC57 plasmids with inserts of the targeted HPV sequences, synthesized by GenScript Corp. (Piscataway, NJ, USA), were utilized. Positive results were considered when cycle threshold (Ct) values were less than 37 for patient specimens. For the performance of vaginal HPV screening to detect cervical HPV infections, we assessed both Ct < 37 and Ct < 40.

### Cytology

Cytology was performed on cervical pap smears. The cytologist (BNZ) assessed the presence of ectocervical and endocervical cells. The cytological diagnoses were performed in accordance with the 2014 Bethesda System and included the following classifications: Negative for squamous intraepithelial lesion or malignancy (NILM), atypical squamous cells of undetermined significance (ASCUS), low-grade squamous intraepithelial lesion (LSIL), atypical squamous cells cannot exclude high-grade squamous intraepithelial lesion (ASC-H), atypical glandular cells (AGC), endocervical adenocarcinoma in situ (AIS), high-grade squamous intraepithelial lesion (HSIL), and squamous cell carcinoma (SCC).

### Statistics

The chi-square test was used to compare categorical categories. Bivariate logistic regression analysis was performed, and crude odds ratios (COR) and adjusted odds ratios (AOR) were calculated for explanatory variables correlating with being HR-HPV positive. For concordance we employed Cohen´s kappa statistics where Cohen’s kappa statistics were used where the Cohen’s kappa coefficient (κ) was estimated where κ < 0 = poor, 0–0.20 = slight, 0.21–0.40 = fair, 0.41–0.60 = moderate, 0.61–0.80 = substantial and 0.81–1.00 = almost perfect agreement. For continuous variables standard error of the mean (SEM) or 95% confidence intervals (CI) were indicated. Statistical significance was set at p < 0.05. All analyses were conducted using the Statistical Package for the Social Sciences (SPSS) version 25 (IBM Corp., Armonk, NY, USA).

## Results

Women with cervical and vaginal HR-HPV infections were younger than women without cervical and vaginal HR-HPV infections (*P* = 0.010 and *P* = 0.003, respectively; Table 1). The proportion of women sexually debuting at a younger age than 18 was higher in women with cervical and vaginal HR-HPV infections than in uninfected women (83.4% *vs.* 73.8%, *p* = 0.015, and 82.1% *vs.*74.5%, *p* = 0.045, respectively). The percentage of women with more than one previous sex partner was also higher in the HR-HPV group than in the uninfected group (for cervical infections 73.9% *vs.* 62.1%, *P* = 0.009, for vaginal infections 75.9% *vs.* 60.6%, *P* = 0.001). The proportion of women not using a contraceptive method was lower in women with cervical and vaginal HR-HPV infections than in uninfected women (54.1% vs. 66.0%, *P* = 0.048 and 53.1% vs. 66.9%, *P* = 0.018, respectively). More women replied that they did not know whether they had been previously infected with chlamydia in women with vaginal HR-HPV infections than in HPV-negative women (48.8% vs. 36.2%, *P* = 0.017). To have undergone a previous pap smear test was reported by 57.8% of HPV negative patients and 51.0% of HPV positive patients (*P* = 0.385). The proportion of women with abnormal cytology was higher in women with cervical HR-HPV infections than in uninfected women (24.2% and 12.1%, respectively, *P* = 0.032, Table 1).

**Table 1:** Characteristics of patients (*N* = 413)

Study variables	Cervical HR-HPV				Vaginal HR-HPV		
	**No (n, %)**	**Yes (n, %)**		***P***-**value**	**No (n, %)**	**Yes (n, %)**	***P***-**value**
**Age**				**0.010**			**0.003**
≤ 35	45 (17.6)	39 (24.8)			42 (16.7)	42 (25.9)	
36–45	95 (37.1)	66 (42.0)			90 (35.9)	71 (43.8)	
46–55	89 (34.8)	31 (19.8)			88 (35.0)	32 (19.8)	
> 55	27 (10.5)	21 (13.4)			31 (12.4)	17 (10.5)	
**Education level**				0.869			0.410
Below secondary school	101 (39.5)	63 (40.2)			100 (39.8)	64 (39.5)	
Complete secondary school	104 (40.6)	66 (42.0)			101 (40.2)	69 (42.6)	
More than secondary school	51 (19.9)	28 (17.8)			50 (20.0)	29 (17.9)	
**Occupation**				0.126			0.068
Farming	18 (7.0)	9 (5.7)			17 (6.8)	10 (6.2)	
Civil servant	7 (2.7)	1 (0.6)			7 (2.8)	1 (0.6)	
Business*	101 (39.5)	80 (51.0)			97 (38.6)	84 (51.9)	
Unemployed	95 (37.1)	52 (33.1)			96 (38.3)	51 (31.5)	
Others	35 (13.7)	15 (9.6)			34 (13.5)	16 (9.8)	
**CD4 (cell/µL)**				0.521			0.662
< 200	23 (9.0)	13 (8.3)			23 (9.2)	13 (8.0)	
200–500	106 (41.4)	74 (47.1)			105 (41.8)	75 (46.3)	
> 500	127 (49.6)	70 (44.6)			123 (49.0)	74 (45.7)	
**Viral load (copies)**				0.089			0.111
< 400	251 (98.0)	148 (94.3)			246 (98.0)	153 (94.5)	
400–5000	5 (2.0)	8 (5.1)			5 (2.0)	8 (4.9)	
> 5000	0 (0)	1 (0.6)			0 (0)	1 (0.6)	
**Serious disease**				0.512			0.799
Yes	36 (16.1)	26 (13.8)			32 (15.5)	30 (14.6)	
No	188 (83.9)	163 (86.2)			175 (84.5)	176 (85.4)	
**Smoking habit**				0.212			0.281
No, I have never been a smoker	216 (84.4)	122 (77.7)			208 (82.8)	130 (80.2)	
Yes, I am currently a smoker	13 (5.1)	13 (8.3)			12 (4.8)	14 (8.6)	
I am an ex-smoker	27 (10.5)	22 (14.0)			31 (12.4)	18 (11.2)	
**Alcohol consumption habit**				0.574			0.604
Never/seldom	153 (59.8)	83 (52.9)			150 (59.8)	86 (53.1)	
Once a month	33 (12.8)	23 (14.6)			32 (12.7)	24 (14.8)	
Once a week	23 (9.0)	18 (11.5)			24 (9.6)	17 (10.5)	
Several times a week	47 (18.4)	33 (21.0)			45 (17.9)	35 (21.6)	
**Live births**				0.539			0.429
0–1	59 (23.0)	36 (22.9)			59 (23.5)	36 (22.2)	
2 and above	197 (77.0)	121 (77.1)			192 (76.5)	126 (77.8)	
**Abortions**				0.484			0.076
0–1	220 (85.9)	136 (86.6)			211 (84.1)	145 (89.5)	
2 and above	36 (14.1)	21 (13.4)			40 (15.9)	17 (10.5)	
**Age at first intercourse**				**0.015**			**0.045**
< 18	189 (73.8)	131 (83.4)			187 (74.5)	133 (82.1)	
18 and above	67 (26.2)	26 (16.6)			64 (25.5)	29 (17.9)	
**Number of sex partners**				**0.009**			**0.001**
1 partner	97 (37.9)	41 (26.1)			99 (39.4)	39 (24.1)	
2 and more partners	159 (62.1)	116 (73.9)			152 (60.6)	123 (75.9)	
**Marital status**				0.465			0.162
Is married	23 (9.0)	12 (7.6)			26 (10.4)	9 (5.6)	
Has a partner but not married	38 (14.8)	23 (14.6)			36 (14.3)	25 (15.4)	
Is separated/divorced or widow	67 (26.2)	32 (20.4)			65 (25.9)	34 (21.0)	
Is single	128 (50.0)	90 (57.4)			124 (49.4)	94 (58.0)	
**Ever had gonorrhea**				0.477			0.053
No	179 (69.9)	107 (68.2)			183 (72.9)	103 (63.6)	
Yes	75 (29.3)	50 (31.8)			66 (26.3)	59 (36.4)	
I don´t know	2 (0.8)	0 (0)			2 (0.8)	0 (0)	
**Ever had syphilis**				0.212			0.111
No	198 (77.3)		118 (75.2)		199 (79.3)	117 (72.2)	
Yes	51 (20.0)		38 (24.2)		46 (18.3)	43 (26.6)	
I don´t know	7 (2.7)		1 (0.6)		6 (2.4)	1 (1.2)	
**Ever had chlamydia**				0.267			**0.017**
No	148 (57.8)		86 (54.8)		156 (62.2)	78 (48.1)	
Yes	5 (2.0)		4 (2.5)		4 (1.6)	5 (3.1)	
I don´t know	103 (40.2)		67 (42.7)		91 (36.2)	79 (48.8)	
**Other sexually transmitted infections**				0.524			0.379
No	45 (17.6)		28 (17.8)		40 (19.3)	33 (16.0)	
Yes	211 (82.4)		129 (82.2)		167 (80.7)	173 (84.0)	
**Contraceptive method**				**0.048**			**0.018**
Male condom	13 (5.1)		13 (8.3)		13 (5.2)	13 (8.0)	
Other method than male condom	74 (28.9)		59 (37.6)		70 (27.9)	63 (38.9)	
I am not using a contraceptive method	169 (66.0)		85 (54.1)		168 (66.9)	86 (53.1)	
**Pap smear test**				0.385			0.531
Yes	148 (57.8)		80 (51.0)		144 (57.4)	84 (51.9)	
No	107 (41.8)		76 (48.4)		106 (42.2)	77 (47.5)	
I don’t know	1 (0.4)		1 (0.6)		1 (0.4)	1 (0.6)	
**Cytology**				**0.032**			0.108
Normal	225 (87.9)		119 (75.8)		219 (87.3)	125 (77.2)	
ASCUS	6 (2.3)		7 (4.5)		6 (2.3)	7 (4.3)	
ASC-H	8 (3.1)		6 (3.8)		7 (2.8)	7 (4.3)	
LSIL	4 (1.6)		5 (3.2)		5 (2.0)	4 (2.5)	
HSIL	4 (1.6)		9 (5.7)		4 (1.6)	9 (5.6)	
Inadequate or missing	9 (3.5)		11 (7.0)		10 (4.0)	10 (6.1)	

^*^Business also includes sex work

The prevalence of LR- and HR-HPV infections (Ct < 37 considered positive) in vaginal and cervical samples are displayed in Table 2. Of the studied 413 participants, 39.2% and 38.3% were positive for HR-HPV in the vagina and the cervix, respectively. For LR-HPVs, 4.4% of participants were positive in vaginal samples and 4.8% in cervical samples. The type-specific HPV agreement for all HPV types (both LR- and HR-HPVs) between vaginal and cervical samples was 98.4% (kappa: 0.82 ± 0.02; *p* < 0.0001) and for HR-HPVs 98.3% (kappa: 0.83 ± 0.02; *p* < 0.0001). Screening for vaginal HPVs (both LR- and HR-HPVs) at Ct < 37 gave a sensitivity of 83.5% (95% CI: 78.4–87.7%), specificity of 99.1% (﻿95% CI: 98.8–99.3%), positive predictive value (PPV) of 81.6% (﻿95% CI: 77.0–85.5%) and negative predictive value (NPV) of 99.2% (﻿95% CI: 99.0–99.4%). Screening for vaginal HR-HPVs gave a similar performance (see Table 3). Screening for vaginal HPVs at Ct < 40 performed worse than at Ct < 37 and gave a sensitivity of 81.8% (﻿95% CI: 77.2–85.8%), specificity of 98.3% (﻿95% CI: 98.0–98.7%), PPV of 75.1% (﻿95% CI: 70.9–78.8%) and NPV of 98.9% (﻿95% CI: 98.6–99.1%).

**Table 2:** Prevalence of HPVs in different mucous membranes

	**Yes/No**	**N**	**%**
**Vaginal HR-HPV**	No	251	60.8
	Yes	162	39.2
**Vaginal LR-HPV**	No	395	95.6
	Yes	18	4.4
**Cervical HR-HPV**	No	255	61.7
	Yes	158	38.3
**Cervical LR-HPV**	No	393	95.2
	Yes	20	4.8

*HPV* human papillomavirus, *HR* high-risk, *LR* low-risk

**Table 3:** Accuracy of vaginal screening at different CT values

**All types (Ct < 37)**	**Sensitivity**	**Specificity**	**PPV**	**NPV**
	83.5 (78.4–87.7)	99.1 (98.8–99.3)	81.6 (77.0–85.5)	99.2 (99.0–99.4)
**HR-HPVs (Ct < 37)**	**Sensitivity**	**Specificity**	**PPV**	**NPV**
	84.9 (79.8–89.1)	99.0 (98.7–99.3)	82.2 (77.5–86.1)	99.2 (98.4–99.4)
**All types (Ct < 40)**	**Sensitivity**	**Specificity**	**PPV**	**NPV**
	81.8 (77.2–85.8)	98.3 (98.0–98.7)	75.1 (70.9–78.8)	98.9 (98.6–99.1)
**HR-HPVs (Ct < 40)**	**Sensitivity**	**Specificity**	**PPV**	**NPV**
	81.7 (76.9–85.8)	98.1 (97.7–98.5)	74.3 (70.0–78.1)	98.8 (98.4–99.0)

*Ct cycle threshold, HR-HPV high-risk human papillomavirus, PPV* positive predictive value, *NPV* negative predictive value

The prevalence of different HPV strains in the genital mucous membranes of women is displayed in Table 4. All analysed HPV strains were detected in the cohort. HPV58 (14.3%/13.5%), HPV52 (12.5%/12.4%), HPV51 (12.9%/11.7%) and HPV16 (9.9%/11.3%) were the most common HPV vaginal/cervical HPV infections. For type-specific concordance between vaginal and cervical samples, HPV33 displayed the highest concordance of 99.5% (kappa: 0.93; SEM: 0.05), while HPV6 displayed the lowest concordance of 97.8% (kappa: 0.66; SEM: 0.11). Co-infections by multiple HPVs were common in the cohort and up to seven HPV types were detected in the same participant (Table 5). Of HPV-positive participants, 32.7% and 35.9% were infected with multiple HPVs in the cervix and vagina, respectively.

**Table 4:** Low-risk and high-risk HPV infections in the vagina and the uterine cervix n (%)

HPV Infections (Ct < 37)	Cervix HPV n (%)	Vagina HPV n (%)	Sum (% of all infections)	Concordance (S.E.M)
HPV6	15 (5.6)	12 (4.4)	27 (5.0)	0.66 (0.11)
HPV11	6 (2.3)	7 (2.6)	13 (2.4)	0.77 (0.13)
HPV16	30 (11.3)	27 (9.9)	57 (10.6)	0.87 (0.04)
HPV18	20 (7.5)	19 (7.0)	39 (7.2)	0.81 (0.07)
HPV31	16 (6.0)	15 (5.5)	31 (5.8)	0.77 (0.09)
HPV33	14 (5.3)	14 (5.1)	28 (5.2)	0.93 (0.05)
HPV35	20 (7.5)	21 (7.7)	41 (7.6)	0.72 (0.08)
HPV39	12 (4.5)	14 (5.1)	26 (4.8)	0.76 (0.09)
HPV 45	12 (4.5)	13 (4.8)	25 (4.6)	0.88 (0.07)
HPV51	31 (11.7)	35 (12.9)	66 (12.3)	0.82 (0.06)
HPV52	33 (12.4)	34 (12.5)	67 (12.5)	0.82 (0.05)
HPV56	18 (6.8)	19 (7.0)	37 (6.9)	0.80 (0.07)
HPV58	36 (13.5)	39 (14.3)	75 (13.9)	0.87 (0.04)
HPV59	3 (1.1)	3 (1.1)	6 (1.1)	0.66 (0.22)
All types (Ct < 37)	266	272	538	0.82 (0.02)
HR-HPVs (Ct < 37)	245	253	498	0.83 (0.02)
All types (Ct < 40)	335	365	700	0.77 (0.02)
HR-HPVs (Ct < 40)	311	342	653	0.76 (0.02)

*Ct* cycle threshold, *HPV* human papillomavirus, *HR* high-risk, *S.E.M.* standard error of the mean

**Table 5:** Number of present HPV types in the vagina and cervix

All HPV types	One type	Two types	Three types	Four types	Five types	Six types	Seven types
Vagina N (%)	107 (64.1)	36 (21.6)	12 (7.2)	6 (3.6)	3 (1.8)	2 (1.2)	1 (0.6)
Cervix N (%)	113 (67.3)	33 (19.6)	8 (4.8)	11 (6.6)	0 (0)	2 (1.2)	1 (0.6)

*HPV* human papillomavirus

Women aged 45–55 years had a lower risk of contracting vaginal HR-HPV infections (COR: 0.40 [95% CI: 0.22–0.72], 0.003; AOR: 0.52 [95% CI: 0.23–1.19], *P* = 0.125) and cervical HR-HPV infections (COR: 0.68 [95% CI: 0.20–0.65], *P* = 0.001; AOR: 0.38 [95% CI: 0.16–0.87], *P* = 0.023) compared to women aged 35 or under (Table 6). Debuting sexually at age 18 or older protected against cervical HR-HPV infection in univariate (COR: 0.56 [95% CI: 0.33–0.92], *P* = 0.024) but not in multivariate analysis (AOR: 0.79 [95% CI: 0.44–1.41], *P* = 0.437). Having had more than one sexual partner was correlated with an increased risk of contracting cervical HR-HPV infections (COR: 1.72 [95% CI: 1.11–2.67], *P* = 0.014; AOR: 1.69 [95% CI: 0.97–2.95], *P* = 0.06) and vaginal HR-HPV infections (COR: 2.05 [95% CI: 1.32–3.19], *P* = 0.001; AOR: 1.78 [95% CI: 1.02–3.11], *P* = 0.042). Not knowing if being infected with chlamydia increased the risk of present vaginal HR-HPV infections (COR: 1.73 [95% CI: 1.15–2.60], *P* = 0.008; AOR: 1.65 [95% CI: 1.01–2.69], *P* = 0.046; Table 6).

**Table 6:** Risk of HR-HPV infections in the vagina and cervix mucous membranes

	**HR-Cervical HPV**								**HR-Vaginal HPV**							
	**COR (95% CI)**		**Sig**		**AOR (95% CI)**		**Sig**		**COR (95% CI)**		**Sig**		**AOR 95% CI)**		**Sig**	
**Age**																
≤ 35	**Reference**				**Reference**				**Reference**				**Reference**			
36–45	0.80 (0.47–1.36)			0.415	0.77 (0.41–1.43)		0.414		0.78 (0.46–1.33)			0.380	0.76 (0.41–1.42)			0.403
46–55	0.40 (0.22–0.72)			**0.003**	0.52 (0.23–1.19)		0.125		0.68 (0.20–0.65)			**0.001**	0.38 (0.16–0.87)			**0.023**
> 55	0.89 (0.44–1.83)			0.766	1.40 (0.51–3.83)		0.512		0.54 (0.26–1.13)			0.107	0.65 (0.23–1.85)			0.429
**Education**																
Less than secondary school	**Reference**				**Reference**				**Reference**				**Reference**			
Complete secondary school	1.01 (0.65–1.58)			0.939	0.99 (0.60–1.62)		0.982		1.06 (0.68–1.65)			0.770	0.95 (0.58–1.56)			0.849
More than secondary school	0.88 (0.50–1.53)			0.654	1.40 (0.59–2.18)		0.691		0.90 (0.52–1.57)			0.728	1.16 (0.60–2.24)			0.649
**Occupation**																
Farming	**Reference**				**Reference**				**Reference**				**Reference**			
Civil servant	0.28 (030–2.69)			0.286	0.24 (0.02–2.86)		0.245		0.24 (0.26–2.27)			0.243	0.22 (0.02–2.51)			0.227
Business	1.58 (67–3.71)			0.290	1.09 (0.42–2.81)		0.857		1.47 (0.63–3.38)			0.363	0.91 (0.35–2.35)			0.857
Unemployed	1.09 (045–2.61)			0.838	0.77 (0.29–2.81)		0.602		0.90 (0.38–2.11)			0.815	0.64 (0.24–1.67)			0.368
Others	0.85 (0.31–2.33)			0.763	0.56 (0.18–1.74)		0.322		0.80 (0.30–2.13)			0.656	0.45 (0.14–1.40)			0.170
**CD4 counts**																
< 200	**Reference**				**Reference**				**Reference**				**Reference**			
200–500	1.23 (0.58–2.59)			0.577	1.27 (0.56–2.88)		0.563		1.26 (0.60–2.65)			0.536	1.53 (0.66–3.54)			0.321
> 500	0.97 (0.46–2.04)			0.947	0.86 (0.37–1.96)		0.725		1.06 (0.50–2.22)			0.868	1.08 (0.46–2.50)			0.857
**Viral Load**																
< 400	**Reference**				**Reference**				**Reference**				**Reference**			
400–5000	2.71 (0.87–8.44)			0.085	2.51 (0.72–8.68)		0.146		2.57 (0.82–8.00)			0.103	3.41 (0.95–12.18)			0.059
**Smoking**																
Never	**Reference**				**Reference**				**Reference**				**Reference**			
Ever	1.54 (0.93–2.56)			0.089	1.46 (0.81–2.62)		0.202		1.19 (0.71–1.97)			0.500	1.00 (0.55–1.83)			0.977
**Use alcohol**																
No	**Reference**				**Reference**				**Reference**				**Reference**			
Yes	1.32 (0.88–1.97)			0.169	0.93 (0.56–1.54)		0.792		1.31 (0.88–1.95)			0.181	0.80 (0.48–1.33)			0.396
**Live births**																
0–1		**Reference**				**Reference**				**Reference**				**Reference**		
2 and above		1.00 (0.62–1.61)	0.978			0.99 (0.55–1.79)		0.984		1.07 (0.67–1.72)	0.762			1.39 (0.76–2.54)	0.274	
**Abortions**																
0–1		**Reference**				**Reference**				**Reference**				**Reference**		
2 and above		0.94 (0.52–1.68)	0.844			1.00 (0.52–1.91)		0.988		0.61 (0.33–1.13)	0.120			0.63 (0.32–1.26)	0.196	
**Age at first intercourse**																
< 18		**Reference**				**Reference**				**Reference**				**Reference**		
18 and above		0.56 (0.33–0.92)	**0.024**			0.79 (0.44–1.41)		0.437		0.63 (0.39–1.04)	0.072			1.03 (0.58–1.83)	0.912	
**Number of sex partners**																
1 partner		**Reference**				**Reference**				**Reference**				**Reference**		
2 and more partners		1.72 (1.11–2.67)	**0.014**			1.69 (0.97–2.95)		0.060		2.05 (1.32–3.19)	**0.001**			1.78 (1.02–3.11)	**0.042**	
**Marital status**																
Is married		**Reference**				**Reference**				**Reference**				**Reference**		
Has a partner but not married		1.16 (0.48–2.76)	0.738			0.95 (0.35–2.56)		0.921		2.00 (0.80–5.00)	0.136			1.51 (0.54–4.26)	0.429	
Is separated/divorced or widow		0.91 (0.40–2.06)	0.832			0.90 (0.35–2.30)		0.840		1.51 (0.63–3.58)	0.349			1.68 (0.63–4.52)	0.298	
Is single		1.34 (0.63–2.84)	0.434			0.99 (0.41–2.37)		0.997		2.19 (0.98–4.89)	0.056			1.52 (0.60–3.83)	0.369	
**Ever had gonorrhea**																
No		**Reference**				**Reference**				**Reference**				**Reference**		
Yes		1.11 (0.72–1.71)	0.619			0.97 (0.57–1.65)		0.926		1.58 (1.03–2.43)	0.033			1.52 (0.60–3.83)	0.123	
I don´t know		0 (0-)	1.00			0 (0-)		1.00		0 (0-)	1.00			0 (0-)	1.00	
**Ever had syphilis**																
No		**Reference**				**Reference**				**Reference**				**Reference**		
Yes		1.25 (0.77–2.01)	0.360			0.90 (0.49–1.64)		0.731		1.59 (0.98–2.55)	0.055			0.94 (0.51–1.72)	0.846	
I don´t know		0.24 (0.29–1.97)	0.184			0.27 (0.28–2.70)		0.270		0.56 (0.11–2.85)	0.491			0.49 (0.07–3.16)	0.458	
**Ever had chlamydia**																
No		**Reference**				**Reference**				**Reference**				**Reference**		
Yes		1.37 (0.36–5.26)	0.640			1.08 (0.23–4.93)		0.917		2.50 (0.65–9.57)	0.181			1.64 (0.36–7.48)	0.508	
I don´t know		1.11 (0.74–1.68)	0.586			1.10 (0.67–1.80)		0.687		1.73 (1.15–2.60)	**0.008**			1.65 (1.01–2.69)	**0.046**	
**Contraceptive method**																
Male condom		**Reference**				**Reference**				**Reference**				**Reference**		
Other method than male condom		0.79 (0.34–1.84)	0.598			0.73 (0.27–1.97)		0.543		0.90 (0.38–2.08)	0.806			0.71 (0.26–1.94)	0.508	
I am not using a contraceptive method		0.50 (0.22–1.13)	0.097			0.50 (0.19–1.30)		0.158		0.51 (0.22–1.15)	0.106			0.56 (0.21–1.48)	0.244	

## Discussion

The results of our study show that vaginal and cervical HPV infections are common among WLWH in Rwanda. Vaginal screening is representative of cervical HPV status among the studied participants. Moreover, we confirm previous findings that sexual behaviour and age are factors associated with the contraction of HPV infections [29, 30].

Two out of five participants were infected with HR-HPVs either in the vagina or cervix, which is similar to the HPV prevalence in WLWH in Rwanda reported in a large-scale study from 2016 where 49.2% of WLWH were HPV-positive [8]. The prevalence is, however, higher than the prevalence we observed in another cohort of WLWH and treated at the same clinic at CHUK [7]. This may be due to the fact that the present study relied on qPCR for detection of HPVs, while our previous study relied on the Multiplex Luminex system (Bio-Rad Laboratories, Inc). The higher prevalence of HPV infections may also be influenced by the younger average age of the studied cohort (43 years), which is lower than that of our previously studied cohorts (45–50 years) at the same clinic. [5, 7]. Notably, the type-specific concordance between vaginal HPVs and cervical HPVs aligns with previous studies for example from Denmark, Belgium, Thailand and Zimbabwe [31–34]. As previously demonstrated [35, 36], our results show that young women had a significantly enhanced risk of contracting vaginal and cervical HR-HPV infections. This may be due to younger women having more sex partners than older women, increasing their risk of HPV exposure [37–39]. However, age was an independent factor associated with the risk of contracting HPV. It has been suggested that immune responses in the uterine cervix differ between young and older woman, and that squamous metaplasia in young women may facilitate for HPV entry into the cervical mucosa [40, 41]. We showed that women with more than one sexual partner had a significantly higher prevalence of HR-HPV infections compared to those reporting only one sexual partner. The correlation between the number of sexual partners and the risk of HPV infection is well known [5, 42, 43]. However, we did not observe an impact of marital status on HPV infection unlike findings from women attending a tertiary hospital in South Africa [44].

We did not observe an association between alcohol use or smoking and the presence of genital HPV infections as suggested by previous studies [45–47]. Few studies exist on HIV and risk factors for cervical cancer development in Africa. No association between alcohol and tobacco use and HPV status was shown in a cross-sectional study including 25–55-year-old patients referred for cervical cancer screening in Burkina Faso [48]. In a South African study, alcohol use was shown to constitute a risk factor for development of cervical HPV infections, while this association was not observed for tobacco smoking [49]. A different composition of HPV types in smokers compared to non-smokers among Ugandan WLWH attending ART clinics has been previously demonstrated. The overall prevalence of HPVs was, however, similar between smokers and non-smokers [50]. In a meta-analysis, smoking was shown to be associated with the development of HPV and cervical cancer but not alcohol consumption [51]. Taken together, HIV, alcohol use and smoking constitute risk factors for the development of cervical HPV infections, however, multicollinearity between these factors may demand larger studies than the one we presently conducted.

The association between reporting not knowing of being infected with chlamydia and having a HR-HPV infection in WLWH in Rwanda has been shown in our previous study [7]. Participants´ tendency to frequently respond with "I don’t know" rather than "yes" or “no” to this specific question may be influenced by the stigma surrounding STIs. As we reported in our previous study, we noted that more than half of women attending the HIV clinic at CHUK had undergone a pap smear test, which should be compared to only one out of four of women attending the clinic in 2015 [5, 7]. This is a positive development for healthcare of WLWH in Rwanda.

The prevalence of HPV58, HPV52, HPV51 and HPV16, in both vaginal and cervical samples, mirrors East Africa and Asia [5, 52–55]. These strains have consistently been among the most prevalent HR-HPV types and are strongly associated with cervical cancer. We also observed a high prevalence of co-infections between different HR-HPV strains both in vaginal and cervical samples. The results showed that present HR-HPV infections were significantly associated with cytological changes. The number of cytological changes was lower than anticipated, given the high prevalence of HPV-positive samples. However, the prevalence of cytological changes was similar to the one we observed in the cohort of women we studied in 2015 at the same clinic [7]. In contrast to our previous study, we performed pap smear test instead of ThinPrep pap test, which could have led to a lower sensitivity to detect cytological changes [56]. To note, the prevalence of cervical cytological abnormalities in women attending HIV clinics varies across cross-sectional studies in Africa. For example, in Enugu, Nigeria, the prevalence was 5.7% in women with high CD4 count and 10.2% in women with low CD4 count [57]. The prevalence of cytological abnormalities was 20% in women attending the HIV clinic at the Rwanda Military Hospital, Rwanda, and as high as 46.0% in women attending an HIV clinic in Nairobi, Kenya [58, 59].

Our study demonstrates an almost perfect concordance between vaginal and cervical HPV samples when setting the cut-off for Ct at 37. This finding is consistent with previous research showing that vaginal HPV sampling may serve as a reliable proxy for cervical HPV sampling [5, 60, 61]. Setting the cut-off at 40 reduced the performance of vaginal HPV screening particularly for the PPV. Among HPV types, the concordance between vaginal and cervical HPV status was the lowest for HPV6 and the highest for HPV33. Previous studies show that LR-HPVs have a tropism for vaginal epithelium over cervical epithelium [25, 26]. Nonetheless, we observed the agreement between vaginal and cervical HPV status remained almost perfect, even when including LR-HPVs in the analysis.

The strength of our study is that we were able to study vaginal to cervical HPV status in a unique cohort of Rwandan WLWH. Our study has limitations in that we observed a lower prevalence of cytological changes among the studied participants than anticipated. Moreover, we compared cervical HPV status with vaginal HPV status from samples taken by the medical staff instead of those taken by the patients themselves. However, combined with findings from our previous study, we believe that vaginal self-sampling for HPV may reflect cervical HPV status [5]. Moreover, we relied on self-reported information from participants for other STIs than HPV and HIV.

In conclusion, we show a high concordance between vaginal and cervical HR-HPV status. Vaginal HPV sampling may therefore simplify cervical cancer screening in the future particularly if vaginal self-sampling is employed. In the future, vaginal self-sampling HPV kits could potentially be distributed through health centers and community health workers to improve access for both urban and rural populations in Rwanda. However, transitioning from opportunistic HPV screening to a full-scale HPV testing program requires significant resources, including laboratory expertise and infrastructure. We show that age, marital status and sexual behaviour contribute to genital HPV infections. These results highlight the importance of targeted preventive strategies, including HPV vaccination and safe sexual practices, particularly among younger individuals and those with multiple sexual partners.

## Supplementary Information

Supplementary Material 1. Questionnaire

Supplementary Material 2. Table S1

## Data Availability

Additional data can be obtained by writing a request to the Institute of Clinical Sciences Sahlgrenska Academy at the University of Gothenburg, Medinaregatan 3A SE-413 90 Göteborg, Sweden. Email: klinvet@gu.se.

## Abbreviations

AOR: Adjusted odds ratio
ART: Antiretroviral therapy
AGC: Atypical glandular cells
ASC-H: Atypical squamous cells cannot exclude high-grade squamous intraepithelial lesion
ASCUS: Atypical squamous cells of undetermined significance
CI: Confidence intervals
COR: Crude odds ratio
AIS: Endocervical adenocarcinoma in situ
HSIL: High-grade squamous intraepithelial lesion
HR-HPV: High-risk HPV
HPV: Human papillomavirus
LSIL: Low-grade squamous intraepithelial lesion
LR-HPV: Low-risk HPV
VILI: Lugol’s iodine
NILM: Negative for squamous intraepithelial lesion or malignancy
NPV: Negative predictive value
PPV: Positive predictive value
RT-PCR: Real-time PCR
SCC: Squamous cell carcinoma
SEM: Standard error of the mean
VIA: Visual inspection with acetic acid
CHUK: University Teaching Hospital of Kigali
