# Supplementary material for: High Concordance between Vaginal Samples and Cervical Samples of Human Papillomavirus in Women Living with HIV in Rwanda
Source: BMC Infect Dis. 2025 Apr 15;25:527. doi: 10.1186/s12879-025-10840-7 (PMC12001398; doi:10.1186/s12879-025-10840-7)
Supplement: Supplementary file 1 — Supplementary Material 1. Questionnaire [file 12879_2025_10840_MOESM1_ESM.docx]

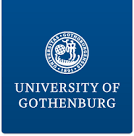
 University of Gothenburg

Gothenburg

Sweden

**Cohort A. Prevalence of HPV co-infections in HIV- and HIV+ Rwandan women**

This questionnaire is part of a research project to investigate how common HPV infection is in the uterine cervix among Rwandan women. Moreover, since HPV may occur in other mucous membranes, such as of the anus and the mouth, we want to assess whether HPV infections exist in these areas too. The Department of Oncology, the Sahlgrenska Academy at the University of Gothenburg, Gothenburg, Sweden, initiated the project. The principal investigator is Associate Professor Daniel Giglio ([daniel.giglio@pharm.gu.se](mailto:daniel.giglio@pharm.gu.se)). Participation in this study is voluntary. Participation is anonymous, and participants will be given a unique code linked to their questionnaire. Coding linking the participant to their questionnaire will only be known to the research group and not given to anyone outside the group./ *Ibi bibazo ni ibijyanye n’ubushakashatsi buzareba ubwiganze bw’agakoko ka HPV gatera uburwayi inkondo yykondo mu bagore babanyarwandakazi, nubwo aka gakoko gashobora kuba mu bindi bice by’yice by byorohereye nko mu nnyo, mu kanwa, turifuza kuzareba niba aka gakoko ka HPV katatera uburwayi no muri ibyo bice bindi. Ubu bushakashatsi bwatangijwe nnatangi rireba ibya cancer muri Sweden, University of Gothenburg, uhagarariye ubushakashatsi aka ari Umwarimu muri iyo Kaminuza Associate Professor Daniel Giglio (*[*daniel.giglio@pharm.gu.se*](mailto:daniel.giglio@pharm.gu.se)*). Kwitabira ubu bushakashatsi ni ubushake. Abazitabira ubushakashatsi amazina yabo ntazagaragazwa, bazahabwa umubare wwubare ubahuza n’nahuza nn babarijweho. Umubare wwubare uhuza uwemeye kuba mu bushashatsi nnshashatsi yabarijweho, uzamenywa gusa nnsa nnwa ryya nnwa gusa n gusa, nta wundi muntu wo hanze yabo uzawumenya.*

Questionnaire identification number/*Umubare uranga ifishi y’ibibazo mu bushakashatsi*:

Participant Identification number in the study/*Umubare uranga uwagize uruhare mu gusubiza:*

Interviewer Name/ *Amazina y’ubaza* ............................................

Interview Date/ *Itariki y’ibazwa*............................................

I will ask some questions that will be recorded in this notebook. I must say that everything you answer will be strictly confidential, and the information gathered from you will be used only in scientific reports, without personal identification. / *Ngiye kukubaza ibibazo maze ibisubizo byandikwe muri aka gatabo.Ndakwizeza neza ko abari abari muri ubu bushakashatsi bose bazagira ibanga ku bisubizo uri butange kandi ko bizakoreshwa gusa mu bijyanye n’intego z’ubu bushakashatsi.*

1. **BACKGROUND INFORMATION/ Amakuru y’ibanze**

**1A. What is your age?** *Ufite imyaka ingahe y’amavuko*?……………….

**1B. Gender/*Igitsina cyawe***A. Male/ *Gabo*
B. Female/ *Gore*

**1C. What is your highest educational level attained?** *Ni uruhe rwego rwanyuma rw’amashuri warangije? (Hitamo igisubizo kimwe)*

A. None/ *Ntiyize*

B. Incomplete primary school/ *Ntiyarangije abanza*

C. Complete primary school/ *Yarangije abanza*

D. Secondary school incomplete/ *Ntiyarangije ayisumbuye*

E. Complete Secondary school/ *Yarangije ayisumbuye*

F. Incomplete University/*Ntiyarangije kaminuza*

G. Complete University/ *Yarangije kaminuza*

H. Others (Specify) / *Ibindi (Sobanura)*

**1D. Occupation/ Umurimo ukora**

A. Farming/ *Umuhinzi mworozi*

B. Civil servant/ *Umukozi wa leta*

C. Business/ *Umucuruzi*

D. Community health worker/ *Akora mu buzima*

E. Unemployed/ *Ntabwo akora*

E. Others (specify)/ *Ibindi (Sobanura)*............................................

**1E. Marital status/ *Irangamimerere***

A. Is married/*Arubatse*

B. Has a male partner but not married/ *Afite umugabo batasezeranye*

C. Has a female partner but not married/ *Afite umugore batasezeranye*

D. Is separated/divorced/ *Yatandukanye nuwo bashakanye*

E. Is widow/ *Ni umupfakazi*

F. Is single (never been married or lived with a partner)/ *Ni ingaragu ntiyigeze ashaka cg ngo* *abane n’umugabo cg umugore*

1. **MEDICAL HISTORY/*AMATEKA Y’UBUVUZI***

**2A. Have you or had any serious diseases in the past? / *Waba warigeze ugira indwara ikomeye mu gihe cyashize?***

A. Yes/ *Yego*

B. No/ *Oya*

**If, yes, please specify/ *Niba ari yego ni iyihe***

Cardiovascular disease/ *uburwayi bw’umutima?*

Cancer/ *Kanseri?*

Rheumatic disease/ *Indwara zo mu ngingo?*

Other serious disease/ *Indi ndwara ikomeye*

**2B. Do you take any medication regularly? / *Hari imiti ufata ku buryo buhoraho?***

A. Yes/ *Yego*

B. No/ *Oya*

**Conventional drugs/ *Imiti ya kizungu*:..……………**

**Traditional drugs/ *Imiti ya gakondo*: …………….**

**2C. Do you have any allergy/hypersensitivity? / *Hari imiti umubiri wawe ugiraho ikibazo?***

A. Yes/ *Yego*

B. No/ *Oya*

If yes, please specify/ *Niba ari yego ni iyihe?* **………………………..**

1. **HABITS/ *IMICO***

**3A. Are you a smoker? / *Unywa itabi?***

A. No, I have never been a smoker/ *Oya, Sindarinywa na rimwe*

B. Yes, I am currently a smoker/ *Yego, Ubu ndarinywa*

C. I am an ex-smoker/ *Narariretse*

**3B. How often do you drink alcohol? / *Unywa inzoga inshuro zingahe?***

A. Never/seldom/ *Nta na rimwe*

B. Once a month/ *Rimwe mu kwezi*

C. Once a week/ *Rimwe mu Cyumweru*

D. Several times a week/ *Inshuro nyinshi mu Cyumweru*

1. **SEXUAL AND REPRODUCTIVE LIVES/ *UBUZIMA BW’IMYOROROKERE***

We are currently examining virus that can be transmitted through sexual contact. Therefore, we would like to ask you some questions about your sexual and reproductive life. / *Turimo gupima agakoko ka virusi gashobora gukwirakwizwa binyuze mu mibonano mpuzabitsina. Turifuza kubabaza ibibazo ku bijyanye n’ubuzima bw’imyororokere yanyu.*

**4A. At which age in years did you have your first sexual intercourse? *Specify age/ ukora imibonanompuzabitsina bwa mbere wari ufite imyaka ingahe? ______***

**4B. How many sexual partners have you had in your life? / *Wakoranye imibonano mpuzabitsina n’abantu bangahe mu buzima bwawe?***

A. 0 partners/ *Nta n’umwe*

B. 1–2 partners/ *Umwe kugera kuri babiri*

C. 3–4 partners/ *Batatu kugera kuri bane*

D. 5–6 partners/ *Batanu kugera kuri batandatu*

E. 7–8 partners/ *Barindwi kugera ku munani*

F. >8 partners/ *Umunani*

**4C. Have you ever been engaged in orogenital sex? / *Waba warigeze ukora imibonano ikorewe mu kanwa no mu gitsina?***

A. Yes/ *Yego*

B. No/ *Oya*

C. I don´t know/ *Simbizi*

**4D. With how many partners have you been engaged in orogenital sex? / Waba warakoranye imibonano ikorewe mu kanwa no mu gitsina n’abantu bangahe?**

A. 1-2 partners/ *umwe kugera kuri babiri*

B. 3-4 partners/ *Batatu kugera kuri bane*

C. 5-6 partners/ *Batanu kugera kuri batandatu*

D. 7-8 partners*/ Barindwi kugera ku munani*

E. >8 partners/ *Umunani*

**4E. How many live births have you had? / *Wagize imbyaro zingahe zabayeho***

Specify number of live births/ *Vuga umubare*__________________________

**4F. How many spontaneous and/or voluntary abortions have you had? / *Waba warakuyemo inda zingahe ku bushake cg bigutunguye?***

Specify number of abortions/ *Vuga umubare*__________________________

**4G. Have you had gonorrhea? / *Waba wararwaye imitezi?***

A. No/ *Oya*

B. Yes/ *Yego*

C. I don´t know/ *Ntabwo mbizi*

**4H. Have you had syphilis? / *Waba wararwaye mburugu?***

A. No/ *Oya*

B. Yes/ *Yego*

C. I don´t know/ *Ntabwo mbizi*

**4I. Have you had chlamydia? / *Waba wararwaye kalamidiya?***

A. No/ *Oya*

B. Yes/ *Yego*

C. I don´t know/ *Ntabwo mbizi*

**4J. Have you had other sexually transmitted diseases? *Waba wararwaye izindi ndwara zandurira mu mibonano mpuzabitsina?***

A. No/ *Oya*

B. Yes/ *Yego*

C. I don´t know/ *Ntabwo mbizi*

If Yes specify/ *If yes specify*……….

**4K. Are you using a contraceptive method now? *If no, go to question 4M.***

A. Yes / *Yego*

B. No / *Oya*

**4L. Can you specify the method (multiple selections can be made)? / *Vuga iyo ukoresha (Ibisubizo byinshi biremewe)***

A. female sterilization/ *Gufunga burundu ku mugore*

B. male sterilization/ *Gufunga burundu ku mugabo*

C. Contraceptive implant/ *Agapira*

D. Contraceptive injectable/ *Urushinge*

E. Contraceptive pill/ *Ibinini*

F. Male condom/ *Agakingirizo*

G. Rhytm method monitoring fertility/ *Kubara*

H. Withdrawal of penis before ejaculation/ *Kwiyakana*

I. Other methods / Ubundi buryo. Specify/ *Buvuge*________________________

J. I am not using a contraceptive method/ *Nta buryo na bumwe nkoresha mboneza urubyaro*

**4M. Have you had a Pap smear test? / *Wigeze ukorerwa ikizamini cya Pap smear?***

A. Yes/ *Yego*

B. No/ *Oya*

C. I don´t know/ *Simbizi*

1. **HIV STATUS/ *UKO UHAGAZE KU BWANDU BWA AGAKOKO ITERA SIDA***

**5A. Do you know your HIV status? / *Uzi uko uhagaze ku bijyanye n’ubwandu bw’agakoko gatera Sida?***

A. Non-infected (negative)/ *Sinanduye*

B. Infected (positive)***/*** *Naranduye*

C. I don´t know/ *Simbizi*

**5B. Are you under antiretroviral treatment*? / Uri ku miti igabanya ubukana?***

A. Yes/ *Yego*

B. No/ *Oya*

**If yes, is it ok that we contact your physician for information on HIV viral load and CD4 count? *Niba waranduye hari icyo bigutwaye tubajije Muganga wawe amakuru ajyanye n’umubare w’udukoko ndetse n’abasirikare ufite mu mubiri?***

A. Yes/ *Yego*

B. Oya/ *Oya*

**Thank you for your time and effort! / *Murakoze ku gihe n’ubwitange bwanyu!***

**To be completed by a physician from patient file / *Biruzuzwa n’umuganga***

Patient’s HIV Clinical staging

**19. CD4 + Count (cells/dl)**

A. < 200

B. 200-500

C. > 500

**20. HIV viral load (copies/ml)**

A. < 400

B. 400-5000

C. >5000

**Thank you for your time and effort! *Murakoze ku mwanya wanyu n’imbarage!***
