# Supplementary material for: High Concordance between Vaginal Samples and Cervical Samples of Human Papillomavirus in Women Living with HIV in Rwanda
Source: BMC Infect Dis. 2025 Apr 15;25:527. doi: 10.1186/s12879-025-10840-7 (PMC12001398; doi:10.1186/s12879-025-10840-7)
Supplement: Supplementary file 2 — Supplementary Material 2. Table S1 [file 12879_2025_10840_MOESM2_ESM.docx]

Table S1. Primers and probes for real-time PCR.

| HPV type | Mix | Oligo type | Oligo sequence |
| --- | --- | --- | --- |
| 16 | 1 | F | TTGCAGATCATCAAGAACACGTAGA |
|  |  | R | CAGTAGAGATCAGTTGTCTCTGGTTGC |
|  |  | P | 5´-FAM-AATCATGCATGGAGATACACCTACATTGCATGA |
|  |  |  |  |
| 6 | 2 | F | RCGGTTYATAAAGCTAAATTGTACGT |
|  |  | R | AGGGTAACATGTCTTCCATGCA |
|  |  | P | 5´-VIC-AAGGGTCGCTGCCTACACTGCTGG |
|  |  |  |  |
| 58 | 2 | F | GGCATGTGGATTTAAACAAAAGGT |
|  |  | R | TCTCATGGCGTTGTTACAGGTTAC |
|  |  | P | 5´-FAM-CACTGCACAGCGCCCTGTCCAA |
|  |  |  |  |
| 11 | 3 | F | GCTTCATAAAACTAAATAACCAGTGGAA |
|  |  | R | GTCAGGAGGCTGCAGGTCTAGTA |
|  |  | P | 5´-FAM-TCCAGCAGTGTAAGCAACGACCCTTCC |
|  |  |  |  |
| 18 | 3 | F | AGAGGCCAGTGCCATTCGT |
|  |  | R | GGTTCTCTGCGTCGTTGGAGT |
|  |  | P | 5´-VIC-TCCTGTCGTGCTCGGTTGCAGC* |
|  |  |  |  |
| 31 | 4 | F | ATTCCACAACATAGGAGGAAGGTG |
|  |  | R | CACTTGGGTTTCAGTACGAGGTCT |
|  |  | P | 5´-VIC-CTCCAACATGCTATGCAACGTCCTGTC |
|  |  |  |  |
| 51 | 4 | F | AAAGCAAAAATTGGTGGACGA |
|  |  | R | TGCCAGCAATTAGCGCATT |
|  |  | P | 5´-FAM-CATGAAATAGCGGGACGTTGGACG |
|  |  |  |  |
| 33 | 5 | F | ATATTTCGGGTCGTTGGGCA |
|  |  | R | ACGTCACAGTGCAGTTTCTCTACGT |
|  |  | P | 5´-VIC-GGACCTCCAACACGCCGCACA* |
|  |  |  |  |
| 35 | 5 | F | TCGGTGTATGTCCTGTTGGAAAC |
|  |  | R | CATAGTCTTGCAATGTAGTTATTTCTCCA |
|  |  | P | 5´-FAM-TGCATGATTACACCTCGGTTTCTCTACGTG |
|  |  |  |  |
| 39 | 6 | F | GCAGGAAGCTATACAGGACAGTGTC |
|  |  | R | CTTGGGTTTCTCTTCGTGTTAGTCT |
|  |  | P | 5´-FAM-CCCGTTTTGTGGTCCAGCACCG* |
|  |  |  |  |
| 52 | 6 | F | GACATGTTAATGCAAACAAGCGAT |
|  |  | R | CATGACGTTACACTTGGGTCACA |
|  |  | P | 5´-VIC-TGTTCAGAGTGTTGGAGACCCCGACC |
|  |  |  |  |
| 45 | 7 | F | GGACAGTACCGAGGGCAGTGTAA |
|  |  | R | TCCCTACGTCTGCGAAGTCTTTC |
|  |  | P | 5´-VIC-CATGTTGTGACCAGGCACGGCA |
|  |  |  |  |
| 56 | 7 | F | GGCATGTGGATTTAAACAAAAGGT |
|  |  | R | TCTCATGGCGTTGTTACAGGTTAC |
|  |  | P | 5´-FAM-CACTGCACAGCGCCCTGTCCAA |
|  |  |  |  |
| 59 | 8 | F | TGTATGGAGAAACATTAGAGGCTGAA |
|  |  | R | TGGACATAGAGGTTTTAGGCATCTATAA |
|  |  | P | 5´-FAM-AGACACCGTTACATGAGCTGCTGATACGC |
|  |  |  |  |
| Beta-globin | 8 | F | GCTCATGGCAAGAAAGTGCTC |
|  |  | R | GCAAAGGTGCCCTTGAGGT |
|  |  | P | 5´-VIC-AGTGATGGCCTGGCTCACCTGGAC |
|  |  |  |  |

F, forward primer; R, reverse primer; P, probe; *, antisense.
